# Brachytherapy for Prostate Cancer: A Systematic Review

**DOI:** 10.1155/2009/327945

**Published:** 2009-09-01

**Authors:** Georgios Koukourakis, Nikolaos Kelekis, Vassilios Armonis, Vassilios Kouloulias

**Affiliations:** ^1^Radiation Therapy Unit, Second Department of Radiology, University Hospital of Athens “ATTIKON”, Rimini 1 Haidari, 124 64 Athens, Greece; ^2^Second Department of Radiology, University Hospital of Athens “ATTIKON”, Rimini 1 Haidari, 124 64 Athens, Greece; ^3^6th Oncologic Deparment of IKA, Asopiou 4, Alexandras Avenue, 11474 Athens, Greece

## Abstract

Low-dose rate brachytherapy has become a mainstream treatment option for men diagnosed with prostate cancer because of excellent long-term treatment outcomes in low-, intermediate-, and high-risk patients. To a great extend due to patient lead advocacy for minimally invasive treatment options, high-quality prostate implants have become widely available in the US, Europe, and Japan. High-dose-rate (HDR) afterloading brachytherapy in the management of localised prostate cancer has practical, physical, and biological advantages over low-dose-rate seed brachytherapy. There are no free live sources used, no risk of source loss, and since the implant is a temporary procedure following discharge no issues with regard to radioprotection use of existing facilities exist. Patients with localized prostate cancer may benefit from high-dose-rate brachytherapy, which may be used alone in certain circumstances or in combination with external-beam radiotherapy in other settings. The purpose of this paper is to present the essentials of brachytherapies techniques along with the most important studies that support their effectiveness in the treatment of prostate cancer.

## 1. Introduction

Localized prostate cancer can be cured by a variety of treatment options with the standard approaches being radiotherapy (RT) and radical prostatectomy. RT represents an alternative to surgery since this approach may provide excellent results in patients well selected for the procedure [1, 2]. Nevertheless, in order to achieve optimal rates of disease control, research has shown that a sufficiently high RT dose must be administered [3–5]. 

The two main modalities of administaring radiotherapy are external X-ray beams generated by a linear accelerator [4], known as external-beam radiotherapy and the implantation of radioactive sources directly into the tumor, known as interstitial brachytherapy. In contrary to external-beam RT, in interstitial brachytherapy, the radiation does not pass through superficial tissues to reach an internal target. Far from it, the radioactive source (i.e., seed) in interstitial brachytherapy releases most of its dose close to its location. External-beam RT and interstitial brachytherapy can be used alone or in combination to take advantage of their unique attributes.

Permanent implantation and temporary implantation represent the two main categories of interstitial brachytherapy. In permanent implantation, the radioactive sources remain in the tissues, whereas in temporary implantation, the radioactive sources are removed after the desired radiation dose is achieved. Brachytherapy can also be amply depicted by the rate at which the dose is delivered, known as the dose rate. The International Commission on Radiation Units & Measurements refers to a dose rate of 40 to 200 cGy per hour (cGy/h) as a low dose rate (LDR), 200 to 1200 cGy/h as a moderate dose rate, and greater than 1200 cGy/h as a high dose rate (HDR) [6]. 

Either permanent or temporary radioactive source implantation can be used in brachytherapy for patients with prostate cancer, with the dose given at either a very LDR or an HDR. Permanent interstitial brachytherapy for prostate cancer, commonly referred to as prostate seed implantation, uses either iodine 125 (^125^I) or palladium 103 (^103^Pd) as radioactive sources for cases involving very LDR (i.e., <40 cGy/h). Contrary, HDR brachytherapy for prostate cancer uses the temporary placement of a high-activity iridium 192 (^192^Ir) radioactive source, which delivers a high radiation dose over a short period.

The purpose of this paper is to present an overview of the use of brachytherapy as a curative treatment for patients with localized prostate cancer. The results of LDR brachytherapy in patients with low-, intermediate-, and high-risk prostate cancer along with HDR brachytherapy when used alone or as a high-dose supplement combined with external-beam RT for these patients are described. We also describe the method of administration of LDR and HDR brachytherapy, the potential advantages and disadvantages of HDR compared with other forms of brachytherapy, and the directions that this form of treatment may take in the future as its use is further investigated.

## 2. Materials and Methods

### 2.1. Identification of Eligible Studies

We searched MEDLINE and the Cochrane Central Register of Controlled Trials (last search on December 2008) using combinations of terms, such as prostate cancer, brachytherapy, low-dose rate, high-dose rate, and efficacy. We considered all English language controlled trials providing information about the effectiveness of low-dose or high-dose-rate brachytherapy on cancer treatment of prostate, adverse profile effects, and future directions of ongoing research as eligible.

### 2.2. Data Extraction

We extracted information from each eligible study. The data recorded, included author's name, year of publication, number of patients included in the study, combination(s) of treatment used (brachytherapy alone or in combination with external radiation therapy), percentage overall response, median time to progression, and median survival.

## 3. Implant Techniques LDR

### 3.1. Real Time

The real-time method of brachytherapy technique for prostate cancer was developed in 1990 by physicians at the Mount Sinai Medical Center. This technique is heavily based on detailed clinical knowledge of the transverse and sagittal ultrasound anatomy of the prostate gland. According to the original inception of this method, an activity per volume table (nomogram) is used to find the proper amount of activity for the seeds to be implanted. Reliant on the concepts formulated by Patterson and Parker, a peripherally weighted implant can be completed by following a relatively accurate set of guidelines [4]. 

The first step is determination of prostate volume by applying an ellipsoid formula (height *χ* width *χ* length *χ* 0.52). This volume is used to determine the number of seeds and total activity ordered for the patient by referring to a look-up table. In the operating room, the prostate volume is recalculated using step-section planimetry at 5-mm intervals from base to apex. Three longitudinal measurements (anterior, middle, and posterior) of the prostate are made in the midline to find the average length of the gland; this important step serves as a general guide for the number of seeds to be placed within the periphery and interior of the gland. The suggested seed activities for both I-125 (range 0.3–0.6 mCi) and Pd-103 (1.5–3 U) are titrated as such to give a continuous isodose line with each other if placed no further than 1 cm apart. Therefore, a prostate length of 3 cm will require 4 seeds, 1 at both the apex and base and 2 in the middle. A 4-cm length will require 5 seeds and so forth. The number of peripheral needles is determined by taking a circumferential measurement at the prostate's greatest transverse diameter. If the circumference is 12 cm, then at least 12 needles should be used. The final decision on the number of needles and spacing between needles and seeds will be somewhat dependent on the activity per seed selected. A higher activity will allow greater spacing (and therefore fewer needles and seeds) but at a cost of needing to be more conservative with proximity to the urethra and rectum. These simple measurements, in addition to referencing the look-up table, allow one to have a reliable road map for the seed implant without the use of a computer-mediated plan. In addition, it allows the implant team to work from the same set of reproducible assumptions to evaluate new technologies or software innovations [7, 8].

The implantation is purposively divided into an initial peripheral and subsequent interior phase. Placing the peripheral needles only greatly improves the imaging of the anterior needles and seeds. As this technique is highly dependent on direct visualization of the position of each needle and seed, any interference caused by the interior needles could contribute to an inferior dosimetric outcome. The goal of the interior needle and seed placement is to deliver the dose to the base and apex of the gland, to provide dose escalation if desired, and to supplement any “cold” areas not covered adequately by peripheral seed placement.

The initial phase consists of peripheral needle placement (usually 12–18 needles) just inside the prostate capsule, with approximately 1-cm spacing, using the greatest transverse image of the prostate. During this process, it is important, although not imperative, that bilateral needle symmetry is achieved within the prostate. Although the length of the prostate gland and the number of needles required are known, the precise number of seeds required may need to be adjusted on the basis of the evaluation of the intraoperative treatment plan, which is developed in tandem with placement of the peripheral needles. When centers are first starting utilization of this technique, an even loading of the peripheral needles from base to apex as identified on the sagittal image, which depicts the entire length of the Foley catheter, would be recommended. For centers with experience, integration of the treatment planning computer into the determination of precise needle and seed placement can allow for extremely conformal implants that simultaneously deliver an oncologically optimal dose to the entire gland while avoiding hot spots near the urethra. From a technical standpoint, it is important to also remember that the posterior needles should be placed well beyond the prostate capsule into the prostate parenchyma, at least 5 mm from the capsule and/or 8 mm from the inner rectal mucosa. This will assure that the rectal dose is well within the acceptable range. Generally, 75% of the seeds are placed in the periphery, in accordance with the principles of Paterson and Parker. If one uses an intraoperative treatment planning software system, it is very common, especially in smaller glands <35 cm^3^ for 80%–85% of the seeds to be deposited in the periphery of the gland. After insertion of peripheral needles to take into account the effect of edema, prostate deformation, and precise needle position, the images are again recaptured in the treatment planning system and the initial plan is re-evaluated in light of the new position of the prostate. This re-evaluation serves as an additional opportunity to optimize the implant dosimetrically and to critically evaluate peripheral needle distribution. The radioactive sources are placed individually using the sagittal setting of the probe. It is important for the brachytherapist to identify prostatic anatomy before the placement of the sources by referring often to the midline sagittal image to ensure that probe movement and prostate movement are properly accounted for as the sources are placed throughout the peripheral needles. In addition, during this process the exact seed position is mapped by the dosimetrist using the real-time treatment planning software. If a seed slips or clumps, this event is accounted for and its consequences can be evaluated and adjusted for during the remainder of the implant procedure [9]. For source placement, the Mick applicator (Mick TP-200; Mick Radionuclear Instruments, Mount Vernon, NY) is used. It is important to remember that the prostate is a three-dimensional object in terms of its relations to the bladder, urethra, and rectum. The Mick applicator allows the operator the freedom to place seeds closer together or farther apart from each other as required by an individual's anatomy. This is particularly important for insertion of the peripheral apical seeds. Here the prostate anatomy is best visualized by sagittal imaging, and a mechanistic approach is best avoided to ensure that seeds are not placed into the periprostatic tissue, which at this point consists largely of perirectal musculature. In addition, the treatment planning system allows the brachytherapist to judge in 3 dimensions where he or she is at in relation to the urethra throughout the procedure. For order of needle implantation, it is best to be consistent in approach to allow the dosimetry team to follow the progress of the implant accurately. Generally the needles furthest from the probe are the most difficult to visualize and should be implanted first, with progression toward the posterior needles near the probe [10]. 

After the entire periphery is implanted, insertion of interior needles is then undertaken, with the remaining 25% of total activity implanted. This is where the treatment planning computer is particularly important because it allows another opportunity to ensure the dosimetric quality of the implant. In addition, one can often test new dose distributions by varying needle location to best fit the unique characteristics of the implant to this point. Usually 4–7 interior needles are used, located at least 5 mm away from the urethra. The purpose of the interior seeds is to adequately cover the base and apex and not necessarily to provide a high amount of radiation to the center. For centers that are beginning an implant program, we advocate internal needle placement in a U-shape around the urethra. As one gains confidence in the procedure and the use of intraoperative treatment planning software, the inner needle distribution can be more variable and continue to fulfill the planned dose constraints. In addition, at this point the intraoperative dosimetry system may be used to rationalize the use of fewer seeds than originally suggested by the nomogram. It is important to always place at least one seed at the apex and at the base, regardless of what the intraoperative software suggests to ensure adequate 30-day postimplant dosimetry [11–14]. 

Patients with biopsy-confirmed seminal vesicle involvement and negative nodal involvement should have vesicles implanted. Deposition of seeds is accomplished through the peripheral needles or through 4-5 additional needles that are placed in the seminal vesicles after removal of the interior ones. The seeds are placed in the anterior and posterior walls to ensure that the prescription dose cloud covers at least the proximal half of the seminal vesicles [15, 16]. 

When all seeds are implanted, a dynamic cystogram under fluoroscopy is performed to exclude the possibility of seeds placed in the bladder or in the urethra. If present, these can be removed before the patient is taken to the recovery room.

### 3.2. Preplan

After the introduction of image-guided seed deposition with the use of axial transrectal ultrasound by Holm, physicians at the Seattle Prostate Institute refined this original technique by developing the preplanned method of prostate brachytherapy in the mid 1980s [17]. According to this method, a plan is created by the physics staff a few days before the implant by using the transverse transrectal ultrasound images taken up in the office. The patient is similarly positioned in the operating room to duplicate the preplan, the predetermined coordinates are identified, and preloaded needles are then placed.

The plan starts with a volume study of the prostate in the examination office, where transverse images are generated at 5-mm intervals and carefully outlined with a light pen. Then each of the images is entered into the treatment planning computer software that generates a three-dimensional model of the gland and calculates the position of each seed into the prostate with dose designation. Finally, this plan is used in the operating room where physicians attempt to put the patient in the same position as when the preplan was created, by meticulous duplication of external set-up parameters such as hip and knee angles. Needles are preloaded with spacers and are then inserted by the use of a template through the perineum in the prostate. The transrectal ultrasound probe is not used to direct the seed placement but is used to assist in the recreation of the preplan and assure that the needles are positioned in the predetermined locations [18, 19]. The implant begins anteriorly and proceeds posteriorly. Each needle is inserted into its preplanned grid location and then is carefully withdrawn, keeping the obturator stationary for the entire row of alternated seeds to be placed in the predetermined position. Loose seeds can also be placed using the Mick applicator, the clinician should identify a potential deficit not encountered by the preplanning team. When all needles are inserted, a cystoscopy is performed to identify whether any needle was placed in the urethra or bladder. If so, the preloaded strand is removed and reloaded in the needle for repeated insertion. In their early implantations the Seattle group used uniform placing of seeds throughout the prostate. Later, peripheral deposition was used to avoid high doses to the central part of the gland. In addition, the initial ultrasound probes did not allow for a biplanar view, which meant that when the technique originated, only the transverse image was available, creating the need to rely upon the preplan and identification of a fixed base point from which to implant all sources. The introduction of the biplanar probe improved identification of the apex and base, resulting in improved coverage of the ends of the gland [20].

## 4. Clinical Results LDR

### 4.1. Patients with Low-Risk Prostate Cancer

Patients with low-risk prostate cancer are particularly well suited for low-dose rate brachytherapy. Although various brachytherapy regimens, including implant alone, implant plus hormonal therapy, combined implant, and external-beam radiotherapy (EBRT), have been used for patients with low-risk cancers, it has been the consensus of most brachytherapists, as well as the American Brachytherapy Society, that low-dose rate brachytherapy alone is the optimal regimen to maximize cancer control while minimizing morbidity (Table 1). Of the 7 series listed in the table with 10 years of followup, the rate of durable biochemical control ranges from 87% to 94%.

### 4.2. Patients with Intermediate-Risk Prostate Cancer

For patients with intermediate-risk prostate cancer, generally those with a Gleason score of 7, a PSA value 10, or a palpable stage T2b tumor, many practitioners have added either hormonal therapy or EBRT to confer a high cure rate. At Mount Sinai, the following treatment algorithm has evolved. The preferred treatment currently for intermediate-risk prostate cancer is the combination of neoadjuvant antiandrogen therapy for a duration of 3 months, followed by a prostate seed implant to a full dose. This regimen has been shown to improve outcomes compared with those with brachytherapy alone. An alternative option is to combine a partial-dose brachytherapy implant with supplemental EBRT to 45 Gy. Generally, at approximately 7 or more years, the reported biochemical control rate ranges from approximately 70% to 95% (Table 2). There is certainly also heterogeneity in this group of patients based upon the definitions of intermediate risk used as well as the volume of cancer as determined by a pretreatment biopsy. With longer followup and improved staging (such as using percent of biopsy involved with the tumor), a brachytherapist should be able to further identify patients with more advanced intermediate-risk features and determine more precisely which patients would benefit from the addition of EBRT to the prostate only or to the pelvis with or without concurrent adjuvant hormone therapy. This understanding has the potential to bring all treated series to an 80%–90% freedom from biochemical failure rate at and beyond 5 years minimum followup.

### 4.3. Patients with High-Risk Prostate Cancer

From the early inception of treating prostate cancer with brachytherapy, it became known that patients with high-risk disease faired poorly when treated with a seed implant alone [42, 43]. This knowledge led to the practice of combining brachytherapy with EBRT to treat these patients. This approach has resulted in excellent disease control rates (Table 3). At Mount Sinai, using an approach that involves 9 months of hormonal therapy, 103-Pa brachytherapy and external-beam irradiation, the 7-year biochemical control rate was 83% for 360 patients with high-risk prostate cancer [43]. Dattoli et al. [44] reported on 243 patients with high-risk disease treated with combination therapy and showed an 80% biochemical control rate at 13 years. These excellent rates compare favorably to those with radical prostatectomy, especially when one focuses on the subset of patients with high-grade tumors (Gleason score 8–10). At Mount Sinai patients with a Gleason score of 8–10 had a 77.5% freedom from PSA failure (FFPF) rate at 7 years [43]. This appears to be superior to the 10% to 39% rate found alone after radical prostatectomy [47, 48].

## 5. Implant Techniques HDR

### 5.1. Advantages of HDR Brachytherapy

When compared to LDR seed brachytherapy HDR brachytherapy has a number of advantages. Generally the advantages may be considered in three areas, the practical, physical and biological. The practical advantages are self-evident in that there are no free live sources used, no risk of source loss and, since the implant is a temporary procedure, following discharge no issues with regard to radioprotection. Furthermore, it maximises the use of existing facilities. Most radiotherapy centres possess an HDR iridium afterloading machine for other purposes, which makes the procedure cost effective. The physical advantages of temporary HDR brachytherapy for the prostate relate to the ability to place afterloading catheters, not only within the prostate capsule but also in the extraprostatic tissues, bladder base, and seminal vesicles. As a result, more advanced cases can be treated successfully with adequate coverage of extracapsular and seminal vesicle tumour. The procedure in which the clinical target volume (CTV) is defined after implantation enables individualisation of dosimetry according to the potential sites of actual and microscopic tumour. The calculation of dosimetry defined by the source dwell positions within each catheter immediately prior to radiation exposure means that accurate measures of both tumour dose and dose to organs at risk can be relied upon. Furthermore the implant procedure prevents organ motion and therefore there is no need for an additional margin expanding the CTV to the planning target volume (PTV). The biological advantage of HDR brachytherapy relates to the ability to deliver intermittent high dose per fraction radiotherapy safely and conformally to the defined PTV. There is now extensive literature supporting the concept that the radiobiological response of prostate cancer cells is predominantly described by a survival curve with a low *α*/*β* ratio. The actual figure remains a matter of some debate but there is general consensus, it is well below five and possibly as low as two or three with the extreme estimates as low as 1.5 [49]. The implication of this is that high dose per fraction delivery of radiotherapy will be biologically more efficient than either conventional external-beam radiotherapy delivered in 2-3 Gy fractions or LDR seed brachytherapy. Using a simple biologically equivalent dose (BED) formula without correction for half-life of repair, the dose increments obtained using HDR boost schedules after 45 Gy in 25 fractions with a boost of 16 Gy in two fractions are of the order of 125% compared with an external-beam dose of 74 Gy in conventional fractionation. When considering HDR monotherapy, this increment is over 150% using standard schedules of 36 Gy in four fractions. It is widely accepted that there is a dose response for prostate cancer, particularly bulky more advanced disease, and it can be seen therefore that HDR brachytherapy is the most efficient means of obtaining dose escalation in terms of biological dose.

### 5.2. Indications for HDR Brachytherapy

HDR brachytherapy is delivered in one of two situations, either as a boost following an intermediate dose of external-beam radiotherapy, typically 45 Gy, or as monotherapy delivering the total radiation treatment with HDR brachytherapy. Monotherapy schedules vary from two fractions to nine fractions, the majority of groups use two to four fractions with a total dose of 26–36 Gy. The GEC ESTRO group [50] has published guidelines for patient selection for HDR brachytherapy. These include patients with any PSA level provided that there is no demonstrable metastasis, any Gleason score, and stages T1b to T3b. Exclusion criteria include a volume of more than 60 mL, infiltration of the bladder neck, significant urinary obstructive symptoms or pubic arch interference and patients for whom lithotomy or anaesthesia is not possible.

### 5.3. Procedure

The procedure for HDR brachytherapy is similar to that for LDR seed brachytherapy using the transperineal transrectal ultrasound guided approach. Patients require a spinal or general anaesthetic for the procedure. HDR afterloading catheters are evenly spaced within the CTV. Catheter fixation is achieved using a template fixed to the perineum. Commercially available programmes will now integrate ultrasound images to provide a 3D reconstruction of the CTV for planning, whilst the patient is in the operating room. Alternatively postoperative CT scans taken after recovery from the procedure enable more detailed planning prior to treatment exposure. Verification using catheter measurements, fluoroscopy, and repeat scanning before each fraction is essential as postimplant prostatic oedema and retropubic oedema can alter the relation between the prostate gland, organs at risk, and the implanted catheters. Schedules vary but it is possible to deliver two or three fractions over 36 hours with a single implant procedure.

### 5.4. Dosimetry

Dosimetry is based on defined dwell time positions within each catheter. Modern commercial software programmes allow infinite manipulation and optimisation of dose using 2.5 or 5 mm dwell positions. Dose constraints for the organs at risk, in particular the rectum, urethra and bladder can be defined. For example, in Mount Vernon Cancer Centre United Kindgom for a prescription dose of 8.5 Gy the D_2 cm_^3^ for the rectum is defined at less than 6.7 Gy, and the V8.5 Gy is zero. The urethral D 10% is constrained at less than 10 Gy, the D 30% at less than 9.8 Gy and the V10 Gy to zero. HDR afterloading treatment delivery is simple and well tolerated by the patient. Removal of the implant is similarly achieved without difficulty and with no need for further anaesthesia.

## 6. Clinical Results HDR

### 6.1. Combined Brachytherapy and External-Beam Radiation Therapy

The greatest clinical experience with HDR brachytherapy for prostate cancer involves its combination with external-beam RT. In this context, external-beam RT is used to treat the prostate and the pelvic tissues (e.g., seminal vesicles), in which there may be microscopic deposits of cancer. The standard external-beam RT dose varies somewhat from one medical institution to another, but in the studies included in the current paper, generally 3600 cGy to 5000 cGy was delivered in 20 to 28 daily treatment sessions [51–62]. HDR prostate brachytherapy was used in these studies to deliver an additional 1200 cGy to 3000 cGy to the prostate [51–62]. HDR brachytherapy may be performed before external-beam RT, after its completion, or in the midst of this component of RT. In this setting, HDR brachytherapy is used to deliver a high dose of radiation to the target to improve tumor control without increasing the risk of injury to the surrounding healthy organs. The medical literature reviewed in the current paper collectively included more than 5000 patients who were treated with the combination of HDR brachytherapy and external-beam RT. Most reports describe clinical outcome using freedom from biochemical relapse as a reporting end point (Table 4). As discussed by Demanes et al. [60] these results are comparable to, or better than, results reported with external-beam RT alone [69, 70] with permanent interstitial LDR brachytherapy alone [35, 70] or with the combination of external-beam RT and LDR brachytherapy [70]. Radiation doses used in HDR brachytherapy were initially selected to some extent on a presumption of efficacy and with safety in mind. However, Galalae et al. increased the HDR brachytherapy dose in a stepwise manner in an attempt to identify an optimal dose [54]. Their research demonstrated that a dose of 1650 cGy or greater delivered in 2 sessions, which is now considered a high dose, led to improved results regarding freedom from biochemical relapse. The combination of HDR brachytherapy and external-beam RT appears to be well tolerated by most patients. Severe gastrointestinal adverse events typically occur in less than 1% of patients [52, 55–60] and moderate gastrointestinal adverse events are experienced by approximately 5% of patients [52, 55, 56, 58–60]. Similarly, severe genitourinary adverse events, mainly consisting of urethral stricture responsive to dilatation, are not apt to occur [55–60, 71], whereas mild to moderate genitourinary adverse events occur in approximately 10% of patients [55, 56, 58–60]. Duchesne et al. [72] noted that adverse events resolve in two-thirds of affected patients after combination therapy, so chronic sequelae are decidedly uncommon. Urinary incontinence was noted in less than 4% of patients, mainly occurring only in the setting of previous or subsequent TURP (transurethral prostatectomy). Demanes et al. [60] reported that erectile function is preserved in approximately two-thirds of patients with prostate cancer after combination therapy.

### 6.2. HDR Brachytherapy Alone

High-dose-rate brachytherapy is also used as the sole method of administering RT for prostate cancer without the addition of external-beam RT. This treatment strategy was developed largely independently at several medical centers. Thus, the number of implantation sessions, the number of treatments, and the prescribed dose have varied somewhat. In performing HDR brachytherapy alone, 1 or 2 implantation sessions have been used to deliver 4 or 6 doses of 600 cGy to 950 cGy each, for a total dose of 3800 cGy to 5400 cGy [63–68, 73]. This approach has provided excellent intermediate-term results regarding freedom from biochemical relapse for certain groups of patients with prostate cancer (Table 5) [63–68, 73]. 

These outcomes appear to compare favorably with results of permanent LDR brachytherapy [64, 65, 74] and with results of the combination of HDR brachytherapy and external-beam RT [75, 76]. Nevertheless, the reported patient followup duration after HDR brachytherapy alone has been shorter than that available for patients treated with combined HDR brachytherapy and external-beam RT. Consequently, the favorable results for HDR brachytherapy alone should be considered somewhat tentatively. High-dose-rate brachytherapy is typically well tolerated by patients with prostate cancer, and the rate and severity of adverse events associated with this treatment compare favorably with permanent interstitial LDR brachytherapy [65]. However, approximately one-half to two-thirds of patients treated with HDR brachytherapy experience acute dysuria, urinary frequency and urgency, or urinary retention [65, 68, 73].

The rate of intermittent self-catheterization for urinary retention is less than 5% [63, 65, 67, 68, 73]. Although diarrhea, proctalgia, and hematochezia can occur, these adverse effects are infrequently encountered [63, 65–68, 73]. Acute adverse events are usually mild and resolve spontaneously, but short-term medicinal therapy may improve genitourinary function and patient comfort. Most patients do not have late effects from HDR brachytherapy, but dysuria, urinary frequency and urgency, urinary retention, hematuria, diarrhea, proctalgia, and hematochezia can occur [63, 65, 66, 68]. These effects tend to be mild and resolve spontaneously [65] but patient recovery may require several months. Urinary stress incontinence and urethral stricture are observed in less than 5% of patients treated with HDR brachytherapy alone [63, 65, 66, 68] and erectile dysfunction is estimated to occur in 16% of patients [65].

## 7. Conclusions

Prostate brachytherapy is an excellent treatment modality for localized prostate cancer. The major side effects are temporary urinary symptoms. In the future, we will most probably be able to better inform patients about their specific risks of side effects, thereby decreasing substantially the influence of any given physician's therapeutic bias in the face of several reportedly equivalent therapies.

Recent technological advances in HDR brachytherapy have increased the appeal and application of this approach for patients with localized prostate cancer. Current treatment methods allow administration of a high dose of radiation that tightly conforms to the targeted volume while minimizing radiation exposure to adjacent healthy organs. Because optimized dose distributions are generated before treatment, high-quality treatment can be assured. To date, patient care data suggest that an impressive therapeutic outcome, with a low rate of adverse events, can be achieved with HDR brachytherapy.

However, several issues regarding HDR brachytherapy remain to be adequately addressed. The ideal radiation dose and number of fractions are not yet known because direct comparisons between various treatment regimens are lacking. Ongoing clinical studies are investigating the feasibility of performing a single implantation, during which only 1 treatment is administered in conjunction with a short course of external-beam RT. This approach would reduce health care costs and medical personnel workload, and it would likely improve patient comfort and convenience.

Randomized clinical trials are needed to directly compare HDR brachytherapy with other forms of treatment for prostate cancer, particularly LDR brachytherapy and external-beam RT. Randomized clinical trials are also needed to determine whether androgen suppression should be integrated into the overall treatment strategy for some patients.

## Figures and Tables

**Table 1:** LDR brachytherapy: clinical results for patients with low-risk prostate cancer.

Author	Number of patients	PSA relapse definition	Median followup	Years after diagnosis	% of biochimical free reccurence
Ellis et al. [21]	239	ASTRO	47 MONTHS	7	96
Zelefsky et al. [22]	319	ASTRO	63 MONTHS	5	96
Zelefsky et al. [23]	1444	ASTRO	63 MONTHS	8	82
Block et al. [24]	118	ASTRO	49 MONTHS	5	94.7
Khaksar et al. [25]	146	ASTRO	45 MONTHS	5	96
Guedea et al. [26]	241	ASTRO	30 MONTHS	3	93
Stock et al. [14]	589	ASTRO	4.2 YEARS	10	94
Prada et al. [27]	275	ASTRO	31 MONTHS	5	96
Potters et al. [28]	481	ASTRO-Kattan	82 MONTHS	12	89
Sharkey et al. [29]	1707	ASTRO	—	12	89
Joseph et al. [30]	667	ASTRO	31 MONTHS	8	84.3
Critz and Levinson [31]	1469	>0.2 ng/mL	6 YEARS	10	93
Bladou et al. [32]	177	NOT DEFINED	29 MONTHS	3	98
Battermann et al. [33]	114	ASTRO	48 MONTHS	5	89
D’Amico et al. [34]	196	ASTRO	3.9 YEARS	5	95
Sylvester et al. [35]	73	2 PSA rises	63 MONTHS	10	89
Kwok et al. [36]	41	ASTRO	7 YEARS	5	85
Grimm et al. [37]	125	2 PSA RISES	81 MONTHS	10	87
Wallner et al. [38]	126	>0.5 ng/mL	2.9 YEARS	3	89–91
Martin et al. [39]	273	Houston	5 YEARS	12	90
Merrick et al. [40]	120	ASTRO	31 MONTHS	5	97

**Table 2:** LDR brachytherapy: clinical results for patients with intermediate-risk prostate cancer.

Author	Number of patients	PSA relapse Definition	Median followup	Years after diagnosis	% of biochimical free reccurence
Ellis et al. [21]	239	ASTRO	47 MONTHS	7	87
Zelefsky et al. [22]	47	ASTRO	63 MONTHS	5	89
Zelefsky et al. [23]	960	ASTRO	63 MONTHS	8	70
Khaksar et al. [25]	111	ASTRO	45 MONTHS	5	89
Guedea et al. [26]	119	ASTRO	30 MONTHS	3	88
Stock et al. [14]	318	ASTRO	4.2 YEARS	10	89.5
Potters et al. [28]	554	ASTRO	96 MONTHS	12	78
Sharkey et al. [29]	1707	ASTRO	—	12	89
Joseph et al. [30]	667	ASTRO	31 MONTHS	8	73.9
Critz and Levinson [31]	1469	>0.2 ng/mL	6 YEARS	10	80
Battermann et al. [33]	114	ASTRO	48 MONTHS	5	75
Sylvester et al. [35]	92	2 PSA rises	63 MONTHS	10	77
Koutrouvelis et al. [41]	68	ASTRO	4 YEARS	5	95
Kwok et al. [36]	33	ASTRO	7 YEARS	5	63
Merrick et al. [40]	273	ASTRO	4.7 YEARS	8	94.8

**Table 3:** LDR brachytherapy: clinical results for patients with high-risk prostate cancer treated with combined brachytherapy and androgen deprivation or external-beam radiation therapy.

Author	Number of patients	PSA relapse definition	Median followup	Years after diagnosis	% of biochimical free reccurence
Ellis et al. [21]	239	ASTRO	47 MONTHS	7	72.5
Dattoli et al. [44]	243	>0.2 ng/mL	8.5 YEARS	13	81
Merrick et al. [45]	204	>0.4 ng/mL	7 YEARS	10	86.6
Zelefsky et al. [22]	192	ASTRO	63 MONTHS	8	48
Khaksar et al. [25]	43	ASTRO	45 MONTHS	5	93
Guedea et al. [26]	30	ASTRO	30 MONTHS	3	81
Stock et al. [14]	360	ASTRO	4.2 YEARS	7	83
Copp et al. [46]	93	ASTRO	54 MONTHS	4	77
Potters et al. [28]	418	ASTRO	82 MONTHS	12	63
Sharkey et al. [29]	1707	ASTRO	—	12	88
Joseph et al. [30]	667	ASTRO	31 MONTHS	8	52.6
Critz and Levinson [31]	1469	>0.2 ng/mL	6 YEARS	10	61
Battermann et al. [33]	114	ASTRO	48 MONTHS	5	54
Sylvester et al. [35]	77	2 PSA rises	63 MONTHS	10	47
Koutrouvelis et al. [41]	280	ASTRO	4 .5 YEARS	5	81
Kwok et al. [36]	28	ASTRO	7 YEARS	5	24

**Table 4:** HDR brachytherapy: percentages biochemical free relapse after combined with external-beam radiation therapy according to risk group for prostate cancer patients.

Author	Number of patients	% of biochemical free reccurence according to risk group			Years after diagnosis
		Low risk	Intermediate risk	High risk	
Aström et al. [51]	214	100	100	86	4
Flynn et al. [52]	674	97	92	79	5
Galalae et al. [53]	611	96	88	69	5
Galalae et al. [54]	324	—	85	81	5
Guix et al. [55]	445	—	95	94	5
Izard et al. [56]	165	100	95	67	5
Martinez et al. [57]	207	—	85	75	5
Phan et al. [58]	309	100	100	97	5
Yamada et al. [59]	105	100	98	92	5
Demanes et al. [60]	209	93	82	62	10
Ghilezan et al. [61]	1577	—	88	74	10
Hasan et al. [62]	886	93	92	71	10

**Table 5:** Clinical results after HDR brachytherapy alone for patients with low- and intermediate-risk prostate cancer.

Author	Number of patients	Free biochemical reccurence (%)	Cause specific survival (%)	Local control (%)	Years after diagnosis
Demanes et al. [63]	298	94	100	100	5
Ghilezan et al. [64]	95	98	100	100	5
Grills et al. [65]	65	98	—	—	3
Mark et al. [66]	206	89	—	—	5
Rogers et al. [67]	328	96 low risk 89 intermediate risk	100	—	3
Yoshioka et al. [68]	111	100 low risk 89 intermediate risk	—	100	3
